# Insight on the roles of stringent response, stringent-like response, and general stress response in hyperosmotic shock tolerance in *Escherichia coli*

**DOI:** 10.1128/mbio.02575-25

**Published:** 2025-09-22

**Authors:** Keilen Kelly, Rajeev Misra

**Affiliations:** 1 School of Life Sciences, Arizona State University 7864, Tempe, Arizona, USA; NYU Langone Health, New York, New York, USA

**Keywords:** osmotic stress, stringent response, general stress response, RelA/SpoT, *rpoS*, *rpoB*

## Abstract

Bacteria frequently encounter osmotic stress in a variety of natural environments, and the primary protective systems they use to adapt to such stresses have been well-characterized. The work examining these mechanisms has largely focused on their role in adapting to moderate shock or continuous osmotic stress. However, survival of rapid, severe osmotic stress remains poorly understood. Here, we demonstrate that a pleiotropic mutation, *rpoB58*, in the beta subunit-coding gene of RNA polymerase in *Escherichia coli* confers tolerance to extreme salt stress. We found that the osmotic shock tolerance phenotype of the mutant is independent of the regulators of two major stress responses, the RelA/SpoT-mediated stringent response (SR) and RpoS-mediated general stress response (GSR). Rather, genetic analysis suggests that *rpoB58* partially mimics the effects of these pathways on gene expression. The data show that pre-activation of these stress responses by a constitutively active *relA* allele, overexpression of *relA* or *rpoS*, or environmental adaptation confers extreme osmotic shock tolerance similar to *rpoB58*. These findings suggest that several conditions, including SR pre-activation, GSR pre-activation, stationary phase, and environmental adaptation, cause cells to pre-emptively adopt a stress-tolerant physiological state and may confer tolerance to extreme osmotic shock by a shared mechanism. Furthermore, pre-activation of SR was found to confer osmotic shock tolerance independent of RpoS, indicating that SR has its own unique role in osmotic shock tolerance.

**IMPORTANCE:**

The overlapping regulation and effects of various stress response pathways in bacteria have been a major subject of study for several decades. This work examines the mechanisms by which a laboratory-acquired mutation in the *rpoB* gene conferring antibiotic tolerance also improves salt tolerance in *Escherichia coli*, an important pathogen of the human gut. We demonstrate that the *rpoB* mutation mimics the effects of multiple stress response pathways on gene expression and that pre-activation of these responses is critical for conferring hyperosmotic shock tolerance. These findings significantly advance our understanding of the genetic mechanisms controlling salt tolerance in bacteria and implicate the stringent response as one factor capable of conferring salt tolerance independent of the general stress response. Furthermore, these findings highlight the intricate connections between salt tolerance and other stress response pathways.

## INTRODUCTION

Enteric bacteria like *Escherichia coli* frequently encounter environmental fluctuations in osmotic strength. The semipermeable membrane helps maintain a homeostatic environment within the cell regardless of external conditions. A hyperosmotic environment, in which the environmental concentration of solutes is greater than inside the cell, causes almost instant water efflux and a rapid cascade of physical and chemical changes in the cell. These changes include decreased intracellular water activity, physical crowding of cytoplasmic molecules, reduced diffusion rates, and increased aggregation (1–7). Turgor pressure also drops as the cytoplasm shrinks, and in Gram-negative bacteria, the inner membrane can peel away from the cell wall in an event called plasmolysis (3, 8–10). These changes can disrupt cellular functions and lead to cell death. The rate and magnitude of changes in osmotic pressure affect viability, with more rapid and larger-magnitude shifts causing greater loss of viability (11–13). Furthermore, severe osmotic shifts affect the lipid composition and integrity of the membrane, which have been linked to loss of viability (14, 15).

To mitigate water efflux, osmotically stressed cells accumulate compatible solutes, osmolytes that can be present in large quantities without affecting intracellular chemistry. Compatible solutes are accumulated in two stages, an initial rapid import through constitutively expressed systems and a delayed activation of other pathways (16–23). If osmotic stress is moderate, the compatible solute intake response adequately protects cells until osmo-adaptation pathways can be activated. On the other hand, rapid and transient stress, referred to as osmotic “shock”, occurs too quickly to allow for activation of such pathways, forcing “shocked” cells to rely exclusively on their physiological state at the time of stress.

Interestingly, response pathways associated with other stresses, such as heat, oxidative, and starvation stress, have cross-protective relationships with osmotic stress (24, 25). For example, pre-exposure to sub-lethal osmotic stress improves tolerance to heat and oxidative stress (26–29), and starved cells display better tolerance to heat, osmotic, and oxidative stresses (24, 25). Mutations in the *relA* and *spoT* genes, the products of which control (p)ppGpp synthesis to induce the stringent response (SR), have been associated with altered response to osmotic shock and heat stress (30, 31). Mutations in RNA polymerase (RNAP) that overcome SR deficiency have been shown to improve tolerance against various stresses (31–35). Additionally, (p)ppGpp participates in the regulation of synthesis of the alternative sigma factor RpoS (σ^S^) (36–38). σ^S^ governs the general stress response (GSR), which includes regulation of osmo-responsive genes, stress tolerance genes, and the transition into stationary phase (29, 39–41). Stationary-phase cells have increased tolerance to a variety of physical and chemical stresses, including osmotic stress (24, 25, 42–44).

We previously characterized a novel point mutation in *rpoB,* coding for the β subunit of RNAP, and showed that it downregulates genes in an SR-like manner and upregulates many genes associated with GSR, including osmotic stress (34). We have demonstrated that the mutation’s stringent-like effects on gene expression confer thermotolerance (35). In this work, we characterize the hyperosmotic shock tolerance phenotype conferred by the same mutation. Though mutations in the β and β′ subunits have been reported to confer tolerance to continuous and moderate (0.3–0.6 M) salt stress (45–49), the mutation discussed here is unique in that it is the first point mutation reported that improves viability during severe (1.3 M NaCl) osmotic shock. We examine the mechanism by which the mutation confers hyperosmotic shock tolerance in the context of the relationships between SR and GSR.

## RESULTS

### *rpoB58* confers tolerance to extreme salt shock

**Fig 1:**
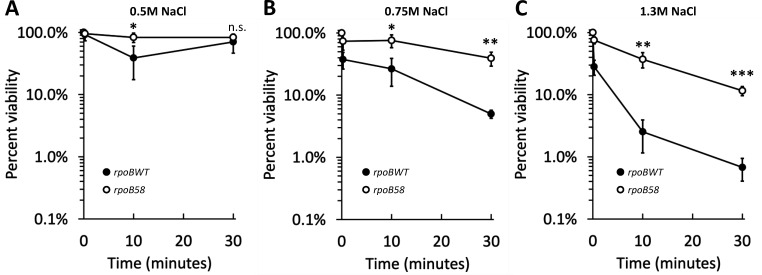
*rpoB58* mutation confers hyperosmotic shock tolerance. Strains RAM3027 (*rpoBWT*, solid symbols) and RAM3028 (*rpoB58*, open symbols) were grown to mid-log (OD_600_ 0.3–0.4) in NB without NaCl before inducing osmotic shock to varying severity with NB + NaCl to 0.5 M (**A**), 0.75 M (**B**), or 1.3 M (**C**). Viability count was determined from each sample just before inducing shock and at 15 s, 10 min, and 30 min after inducing shock. Samples were serially diluted into NB, plated onto LBA, and grown at 37°C overnight to allow colony formation to calculate viability. The experiment was conducted in triplicate. Error bars indicate standard deviation. Statistically significant differences between *rpoBWT* and *rpoB58* data at each time point are marked with asterisks (n.s. not significant, *P < 0.05, **P < 0.01, and ***P < 0.001).

Because *rpoB58* (carrying a G449V substitution in RpoB) was previously shown to constitutively upregulate genes involved in stress-mitigating pathways (34), we hypothesized that *rpoB58* cells adopt a “pre-emptively” stressed state with increased tolerance to multiple stresses. We tested this in the context of salt stress by measuring the effect of hyperosmotic salt shocks on viability. For our assay, exponential phase cultures in salt-free nutrient broth (NB) were mixed with an equal volume of NB + NaCl at two times the desired concentration (e.g., 2.6 M), such that the final mix reached the desired salt concentration (e.g., 1.3 M). Samples were withdrawn over a 30-min period and plated after dilution to obtain viable cell counts. *rpoBWT* (wild-type) cultures experienced a moderate drop in viability immediately upon 0.5 M NaCl shock but almost completely recovered within the 30-min shock. *rpoB58* cultures experienced virtually no loss of viability over the same period (Fig. 1A). However, during 0.75 and 1.3 M shocks, *rpoBWT* cultures experienced immediate and severe decreases in viability and did not recover during the treatment period (Fig. 1B and C). While *rpoB58* cultures experienced some loss of viability during 0.75 and 1.3 M shocks, they maintained significantly higher viability than *rpoBWT* cultures, demonstrating that *rpoB58* confers considerable tolerance to extreme salt shock. Notably, *rpoB58* cultures did not experience dramatic decreases in viability immediately upon higher shocks, but viability dropped over the course of the assay. The immediate difference in viability between *rpoBWT* and *rpoB58* upon shock indicates that *rpoB58* cells pre-emptively adopt a physiological state with improved osmotic shock tolerance.

### Both up-shock and down-shock contribute to the loss of cell viability

The assay described above involved both up-shock (increase in osmotic pressure) and down-shock (decrease in osmotic pressure), because shocked cells were diluted in salt-free NB. We examined whether *rpoB58* improved viability in response to up-shock, down-shock, or both by diluting up-shocked cells in media of varying osmotic strengths (NB without NaCl or with 0.5 or 1.3 M NaCl), reasoning that dilution in higher salt concentrations would reduce or eliminate any effect of down-shock on viability.

**Fig 2:**
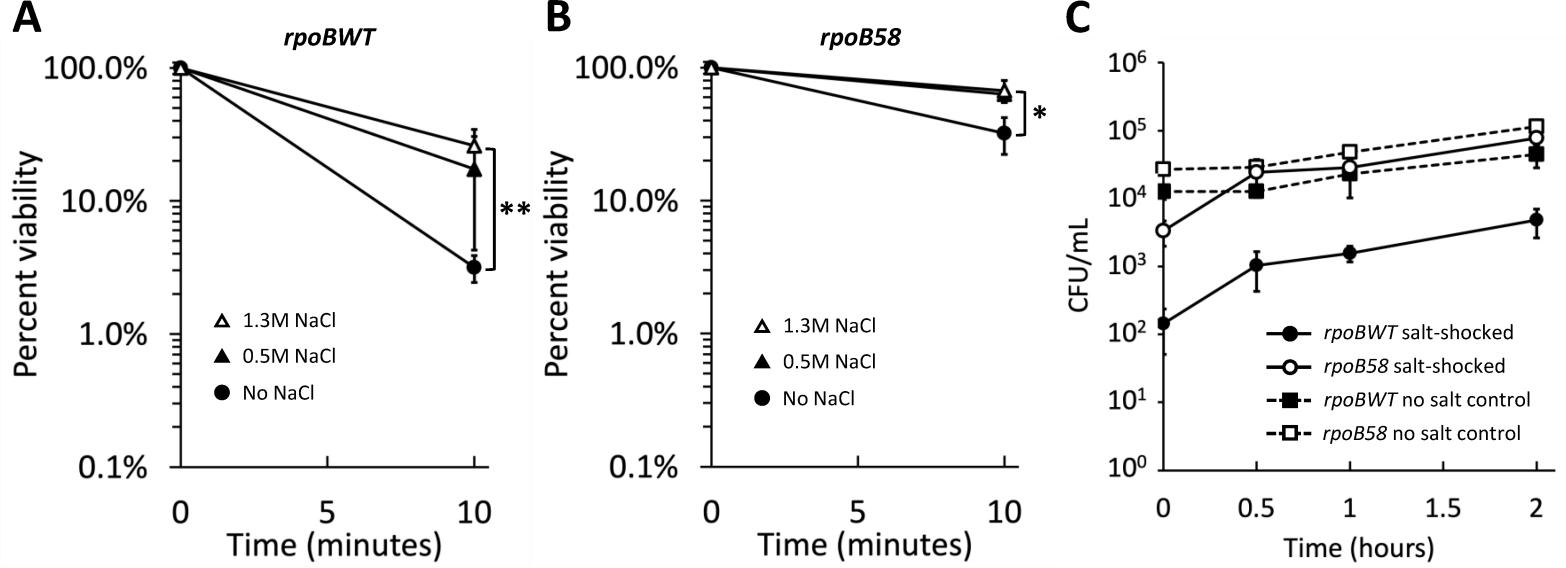
*rpoB58* improves viability following both osmotic up-shock and down-shock but does not affect post-shock rate of recovery. Strains RAM3027 (*rpoBWT*, **A**) and RAM3028 (*rpoB58*, **B**) were grown and shocked with NB + NaCl to 1.3 M, as described in Fig. 1. Just before inducing shock and at 10 min after inducing shock, samples were serially diluted into NB (solid circles), NB + 0.5 M NaCl (solid triangles), or NB + 1.3 M NaCl (open triangles). Dilutions were plated onto LBA and grown at 37°C overnight to allow colony formation to calculate viability. The experiment was conducted in triplicate. Error bars indicate standard deviation. Statistically significant differences between treatment with NB + 1.3 M NaCl (open triangles) and NB without NaCl (solid circles) at the 10-min time point are marked with asterisks (*P < 0.05 and **P < 0.01). (**C**) Mid-log grown cultures of strains RAM3027 (*rpoBWT*, solid symbols) and RAM3028 (*rpoB58*, open symbols) were shocked with NB + NaCl to 1.3 M (circles, solid lines) or not shocked (squares, dashed lines). At 10 min after inducing shock, samples were diluted 10-fold into NB to stop the up-shock effect, incubated for an additional 2 h, and sampled to determine viable cell count. Sampling for viability was done immediately upon dilution to stop up-shock and at 30 min, 1 h, and 2 h after dilution. The experiment was conducted in triplicate. Error bars indicate standard deviation.

Significant killing was observed even without down-shock, indicating that up-shock alone affects viability in both *rpoBWT* and *rpoB58* (Fig. 2A and B). Dilution in higher-salt media reduced killing of both *rpoBWT* and *rpoB58*, indicating that down-shock also contributes to loss of viability. Importantly, the viability of *rpoB58* was consistently higher than *rpoBWT* after shock regardless of dilution medium, showing that *rpoB58* improves tolerance to both up-shock and down-shock.

### Cell growth resumes at the same rate in *rpoBWT* and *rpoB58* after shock

We considered that the differences in cell counts after shock could be explained by different rates of recovery, rather than loss of viability, and we sought to validate our assay by assessing the rate of post-shock recovery. After 10 min of shock, samples were diluted 10-fold to stop up-shock and incubated at 37°C for 2 h, with samples periodically plated to determine the rate at which growth resumed. Figure 2C shows that although *rpoBWT* had much lower viability than *rpoB58* immediately following shock, the remaining viable cells in both *rpoBWT* and *rpoB58* cultures after shock resume growth at similar rates. This indicates that while *rpoB58* confers improved viability following osmotic shock, it does not affect the rate of recovery in surviving cells.

### RpoS (σ^S^) and selected σ^S^-regulated genes are not required for osmotic shock tolerance of *rpoB58*

**Fig 3:**
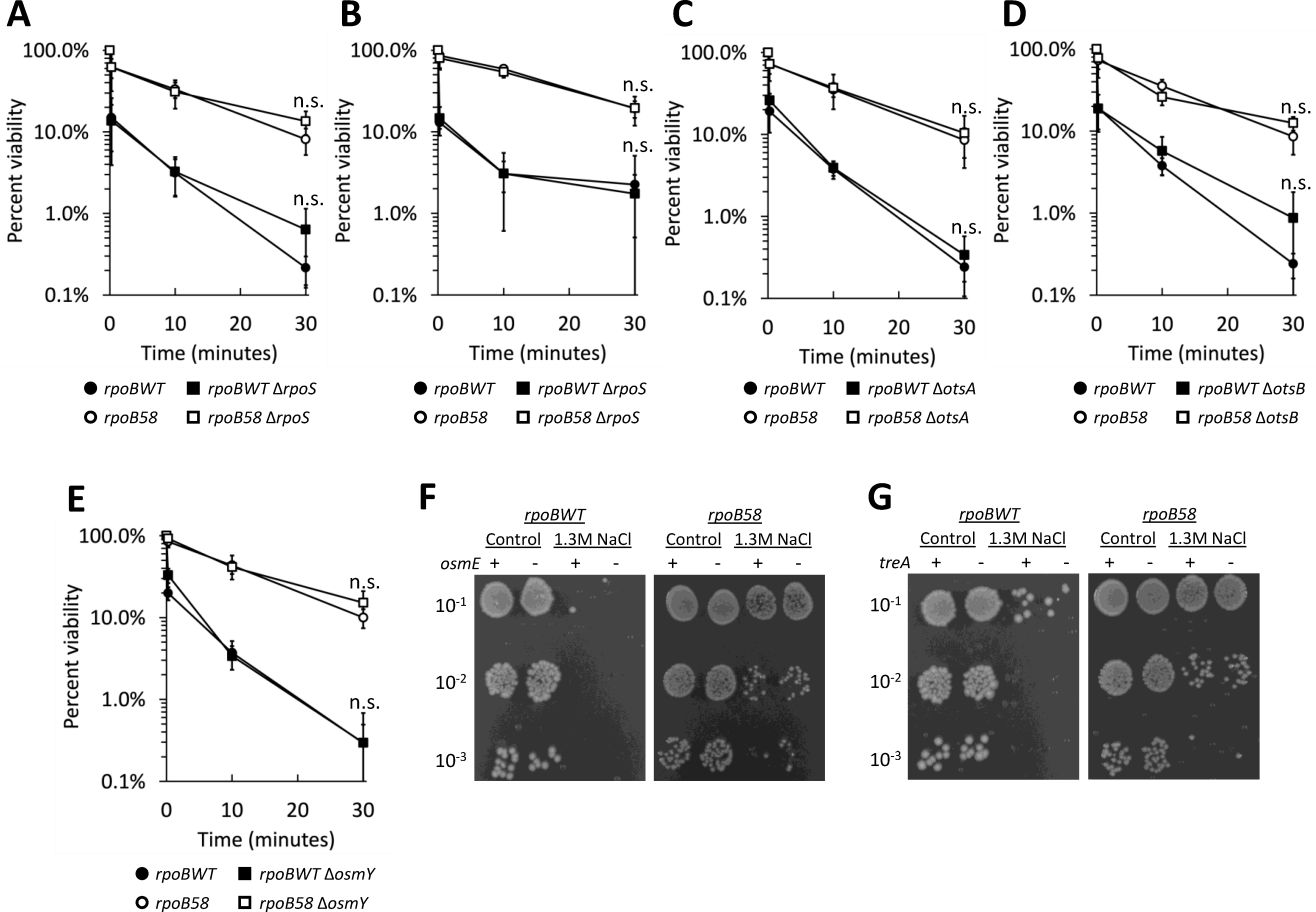
Hyperosmotic shock tolerance is unaffected without RpoS and selected members of the RpoS regulon. (**A and B**) RAM3027 (*rpoBWT*, solid circles), RAM3028 (*rpoB58*, open circles), and their Δ*rpoS::Km* derivatives (solid squares and open squares, respectively) were shocked as in Fig. 1C with 1.3 M (**A**) or 0.75 M NaCl (**B**). (**C**) RAM3027 (*rpoBWT*, solid circles), RAM3028 (*rpoB58*, open circles), and their Δ*otsA::Km* derivatives (solid squares and open squares, respectively) were shocked as in Fig. 1C. (**D**) RAM3027 (*rpoBWT*, solid circles), RAM3028 (*rpoB58*, open circles), and their Δ*otsB::Km* derivatives (solid squares and open squares, respectively) were shocked as in Fig. 1C. (**E**) RAM3027 (*rpoBWT*, solid circles), RAM3028 (*rpoB58*, open circles), and their Δ*osmY::Km* derivatives (solid squares and open squares, respectively) were shocked as in Fig. 1C. Differences between parental strains and derivative deletion strains are not significant at any time point, represented by “n.s.” Error bars represent standard deviation from four (**A**) or three (**B–E**) independent cultures. (**F and G**) Representative images of the 30-min time point dilution plating conducted in the same assay as (**A–E**), but with RAM3027 (*rpoBWT*), RAM3028 (*rpoB58*), and their Δ*osmE::Km* (**F**) and Δ*treA::Km* (**G**) derivatives. Images are representative of three replicates.

We previously showed that *rpoB58* upregulates the σ^S^ regulon, which is controlled by *rpoS* and governs the GSR, transition to stationary phase, and some osmo-regulated genes (34). We wanted to explore whether *rpoS* and σ^S^-regulated genes upregulated by *rpoB58* are involved in shock tolerance. Osmotic shock tolerance was unaffected by *rpoS* deletion in either *rpoBWT* and *rpoB58* at 1.3 M NaCl (Fig. 3A) or 0.75 M NaCl (Fig. 3B). Expression of several σ^S^-regulated and osmo-regulated genes, including *osmE*, *treA*, *osmY*, and *otsBA*, is significantly upregulated in the *rpoB58* mutant (34). However, deletion of these genes individually had no effect on viability in either *rpoB* background (Fig. 3C through G).

### Growth phase contributes to osmotic shock tolerance independent of *rpoS*

**Fig 4:**
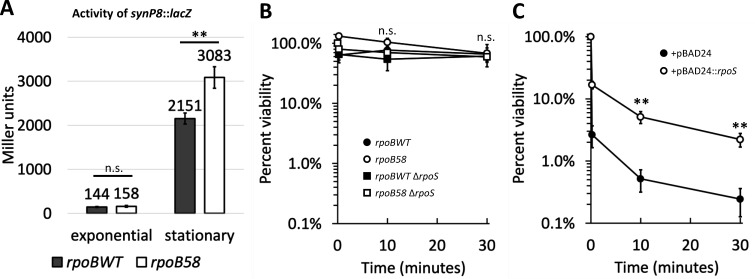
Hyperosmotic shock tolerance is improved by elevated levels of RpoS. (**A**) Strains RAM3188 (*rpoBWT synP8::lacZ*, solid bars) and RAM3189 (*rpoB58 synP8::lacZ*, open bars) were grown in LB to mid-log (OD_600_ 0.3–0.4) or stationary phase (overnight cultures, OD_600_ 4–5) before sampling for β-galactosidase assay. (**B**) Strains as in Fig. 3A were grown to stationary phase before shocking with 1.3 M NaCl. Differences between strains are not significant at any time point. (**C**) Strains RAM3743 (*rpoBWT* + pBAD24, solid circles) and RAM3744 (*rpoBWT* + pBAD24*::rpoS*, open circles) were grown to mid-log (OD_600_ 0.3–0.4) in NB supplemented with ampicillin and 0.1% arabinose before inducing osmotic shock by sudden upshift with NB + NaCl to 1.3 M. Error bars indicate the standard deviation of values from two (**B**) or three (**A and C**) independent cultures. Statistically significant differences are marked with asterisks (n.s. not significant, **P < 0.01).

Until this point, we conducted shock assays using log-phase cultures. Stationary-phase cells are generally more stress tolerant, largely due to σ^S^-regulated effects (50, 51). RpoS activity is quite low during log phase and only rises as cells enter stationary phase (52, 53), as demonstrated by activity of a *lacZ* fusion under the control of a promoter specifically responsive to σ^S^ (54; Fig. 4A). The lack of effect of Δ*rpoS* during salt shock at log phase (Fig. 3A and B) was likely due to low RpoS activity (Fig. 4A), so we investigated whether deletion of *rpoS* would have a stronger effect on osmotic shock tolerance at the stationary phase. Stationary-phase cultures of both *rpoBWT* and *rpoB58* displayed increased osmotic shock tolerance compared to log-phase cultures (Fig. 3A and 4B), indicating that growth phase affects osmotic shock tolerance. *rpoBWT* experienced a greater increase in tolerance from log to stationary phase than *rpoB58*, such that their osmotic shock tolerance was approximately equal at the stationary phase (Fig. 4B). Interestingly, however, the increased osmotic shock tolerance of stationary-phase cells was independent of *rpoS* in both *rpoB* backgrounds (Fig. 4B), demonstrating that the effect of growth phase on osmotic shock tolerance is regulated by an *rpoS-*independent mechanism.

### Overexpression of *rpoS* confers osmotic shock tolerance

We reasoned that *rpoB58* may simply bypass the need for RpoS by mimicking the transcriptional effects of RpoS overexpression, even in the absence of the *rpoS* gene. If so, overexpression of *rpoS* in *rpoBWT* could confer osmotic shock tolerance. We overexpressed *rpoS* from a plasmid in the *rpoBWT* background and found that elevation of RpoS at log phase indeed increased tolerance to 1.3 M NaCl shock (Fig. 4C), suggesting that the osmotic shock tolerance phenotype of *rpoB58* could, in part, be the result of elevated expression of *rpoS* or members of its regulon.

**Fig 5:**
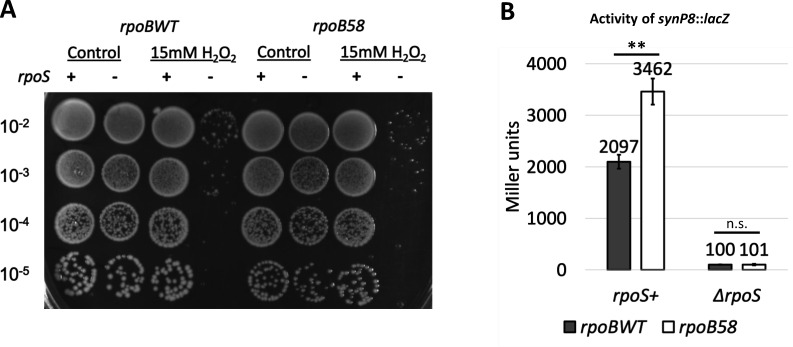
*rpoB58* does not bypass RpoS. (**A**) Strains RAM3027 (*rpoBWT*), RAM3741 (*rpoBWT* Δ*rpoS*), RAM3028 (*rpoB58*), and RAM3742 (*rpoB58* Δ*rpoS*) were grown in LB to stationary phase (overnight cultures, OD_600_ 4–5), washed two times in 0.9% NaCl, and diluted to OD_600_ = 1.0. Samples were then mixed with an equal volume of 0.9% NaCl or 0.9% NaCl + 30 mM H_2_O_2_ for a final solution of either 0 or 15 mM H_2_O_2_. Twenty minutes after adding H_2_O_2_, samples were serially diluted in 0.9% NaCl and plated for colony formation. Image is representative of results from two independent experiments. (**B**) Strains RAM3188 (*rpoBWT synP8::lacZ*), RAM3189 (*rpoB58 synP8::lacZ*), RAM3747 (RAM3188 Δ*rpoS*), and RAM3748 (RAM3189 Δ*rpoS*) were grown in LB to stationary phase (overnight cultures, OD_600_ 4–5) before sampling for β-galactosidase assay. Error bars indicate the standard deviation of values from three independent cultures. Statistically significant differences between *rpoS^+^* and Δ*rpoS* are marked with asterisks (n.s. not significant, ^**^P < 0.01).

To determine whether *rpoB58* altogether bypasses the need for *rpoS*, we examined additional phenotypes of *rpoS* deletion. We found that *rpoB58* still required *rpoS* for H_2_O_2_ tolerance (Fig. 5A), mediated by RpoS-regulated KatE, and for the expression of the RpoS-controlled *lacZ* fusion (Fig. 5B). Therefore, while *rpoB58* bypasses the need for *rpoS* in osmotic shock tolerance by constitutively upregulating certain genes in the RpoS regulon, it does not bypass all *rpoS* phenotypes. Taken together, these data indicate that while RpoS overexpression can confer osmotic shock tolerance in *rpoBWT*, elevated expression of *rpoS* by *rpoB58* is not responsible for the mutant’s shock tolerant phenotype.

### A basal level of SR does not contribute to osmotic shock tolerance

**Fig 6:**
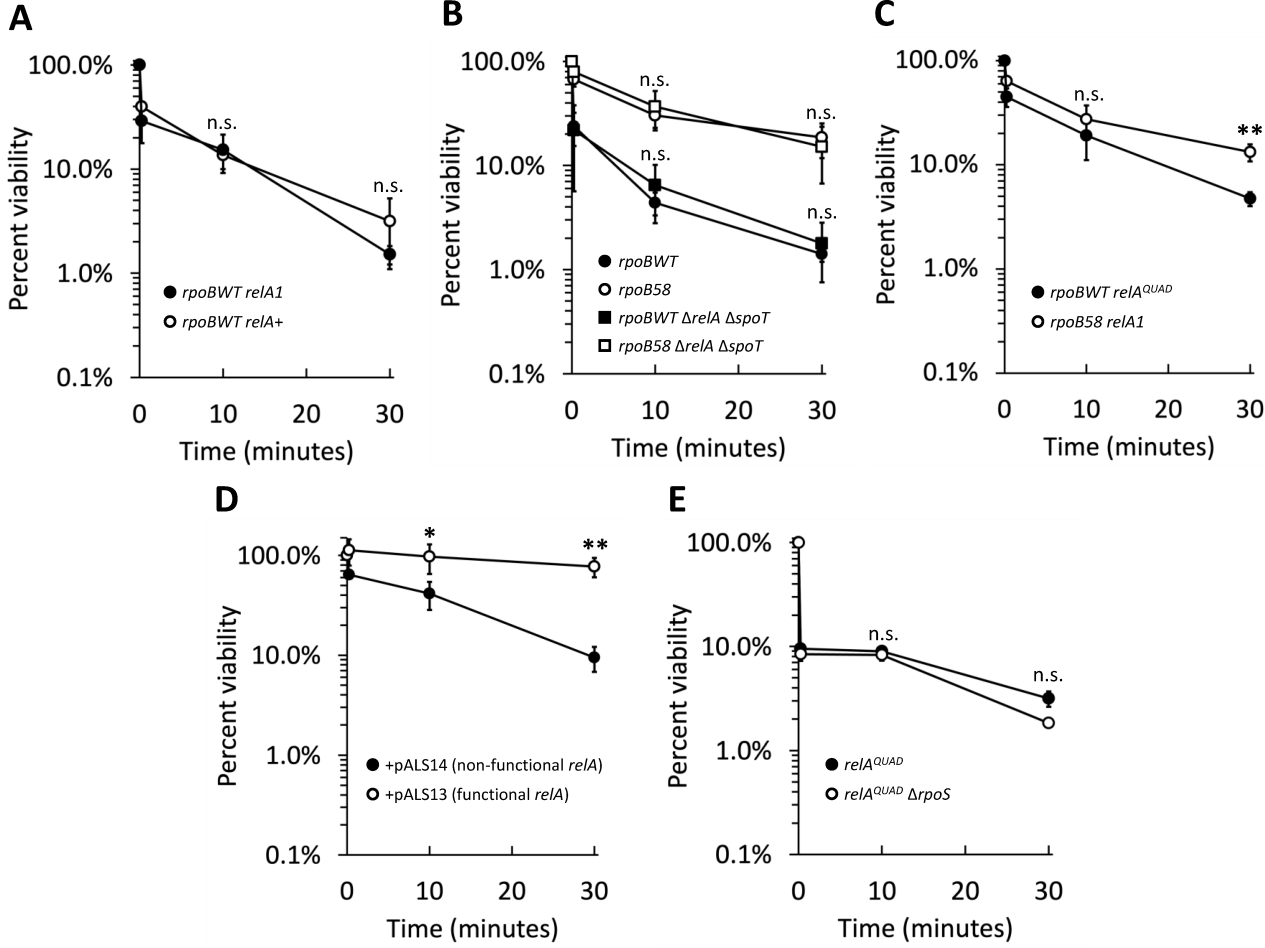
Hyperosmotic shock tolerance is independent of RelA/SpoT but improved with elevated RelA activity. (**A**) Strains RAM3337 (*rpoBWT relA1*, solid circles) and RAM3338 (*rpoBWT relA^+^*, open circles) were shocked as in Fig. 1C. Differences between strains are not significant at any time point. (**B**) Strains RAM3595 (*rpoBWT*, solid circles), RAM3599 (*rpoB58*, open circles), RAM3665 (*rpoBWT* Δ*relA::Km spoT::Cm*, solid squares)*,* and RAM3669 (*rpoB58* Δ*relA::Km spoT::Cm*, open squares) were shocked as in Fig. 1C. Differences between *relA^+^ spoT ^+^* and Δ*relA* Δ*spoT* strains are not significant at any time point. (**C**) Strains RAM3028 (*rpoB58*, open circles) and RAM3486 (*rpoBWT relA^QUAD^*, solid circles) were shocked as in Fig. 1C. Statistically significant differences between strains at each time point are marked with asterisks (n.s. not significant, **P < 0.01). (**D**) Strains RAM3749 (*rpoBWT* + pALS13 [functional *relA* fragment], open circles) and RAM3750 (*rpoBWT* + pALS14 [non-functional *relA* fragment], solid circles) were grown to an OD_600_ of 0.2 in NB supplemented with ampicillin (no added NaCl) before adding IPTG to a final concentration of 10 µM and growing for one additional hour. Induced cultures were then shocked by sudden upshift with NB + NaCl to 1.3 M as described previously. Statistically significant differences between strains at each time point are marked with asterisks (*P < 0.05 and **P < 0.01). (**E**) Strains RAM3491 (*rpoBWT relA^QUAD^,* closed circles) and RAM3751 (*rpoBWT relA^QUAD^* Δ*rpoS*, open circles) were shocked as in Fig. 1C. Differences between *relA^QUAD^* and *relA^QUAD^* Δ*rpoS* strains are not significant at any time point. Experiments (**A–D**) were conducted in triplicate, and experiment (**E**) was conducted in duplicate. Error bars indicate standard deviation.

*rpoB58* was isolated in an MC4100 genetic background, which contains the SR-defective *relA1* allele (55). To test the effect of basal levels of SR in osmotic shock tolerance, we replaced the defective *relA1* allele with the functional *relA*^+^ allele in the *rpoBWT* strain. Cell viability after 1.3 M NaCl shock was similar in both *relA1* and *relA*^+^ backgrounds (Fig. 6A). Because the SR alarmone (p)ppGpp can be produced by both RelA and SpoT (56), we examined whether the complete absence of (p)ppGpp-synthesizing activity in a Δ*relA* Δ*spoT* background would make *rpoBWT* even more sensitive to osmotic shock. The data showed that viability was not affected by the complete absence of (p)ppGpp (Fig. 6B). Taken together, these results indicate that a basal level of SR does not play any significant role in hyperosmotic shock tolerance.

### Pre-activated SR confers osmotic shock tolerance

*rpoB58* appears to partially mimic the effects of constitutively active SR, based on its gene expression profile (34) and ability to overcome the “relaxed” amino acid auxotrophy phenotype of a Δ*relA* Δ*spoT* strain (35). We have termed this SR pre-activation as the “stringent-like response” (S-LR). Viability of the mutant during shock was not affected in the Δ*relA* Δ*spoT* strain (Fig. 6B), indicating that, like the classical “relaxed” phenotype, the S-LR of *rpoB58* can overcome hyperosmotic shock independent of (p)ppGpp.

To better investigate the role of pre-activated SR in osmotic shock tolerance, we examined the effect of two mutant *relA* alleles in the *rpoBWT* background. First, we examined whether a constitutively active variant of *relA*, *relA^QUAD^* (57), protected against salt shock. Figure 6C shows that *relA^QUAD^* significantly improved viability during 1.3 M salt shock, and although the protective effect was not as strong as that conferred by *rpoB58*, this indicates that pre-emptive activation of SR confers osmotic shock tolerance.

We similarly examined the effect of a constitutively active synthetase fragment of RelA on osmotic shock tolerance. Plasmids carrying either a functional *relA* fragment (lacking the autoinhibitory domain and therefore constitutively producing (p)ppGpp) or a non-functional *relA* fragment (58) under the control of an IPTG-inducible promoter were transformed into a *relA*^+^ MG1655 background. Our MC4100 background could not form homogenous colonies after transformation even in the absence of IPTG, presumably due to leaky expression of the functional *relA* fragment, and the MG1655 background was used because it did not show this defect.

After inducing synthesis of the *relA* fragment for one hour by IPTG, strains were shocked with 1.3 M NaCl. In agreement with the effect of *relA^QUAD^*, overexpression of the constitutively active fragment of *relA* conferred increased shock tolerance compared to the inactive fragment (Fig. 6D). Taken together, these data demonstrate that pre-activation of the SR can confer osmotic shock tolerance, whether by mimicking SR through the stringent-like effects of *rpoB58* or by increasing RelA activity via *relA^QUAD^* or overexpression of *relA*.

### RpoS is not required for pre-activation of SR to confer osmotic shock tolerance

Considering the relationship between (p)ppGpp-mediated SR and the activation of RpoS (36, 38) and the ability of RpoS overexpression to confer osmotic shock tolerance (Fig. 4C), we considered that shock tolerance conferred by SR pre-activation could be the indirect result of elevated RpoS levels. We tested this by examining the effect of SR pre-activation in the absence of *rpoS*. We found no significant difference in the shock tolerance of *relA^QUAD^* and *relA^QUAD^* Δ*rpoS* (Fig. 6E)*,* indicating that osmotic shock tolerance conferred by SR pre-activation is RpoS-independent.

### Pre-adaptation confers osmotic shock tolerance

**Fig 7:**
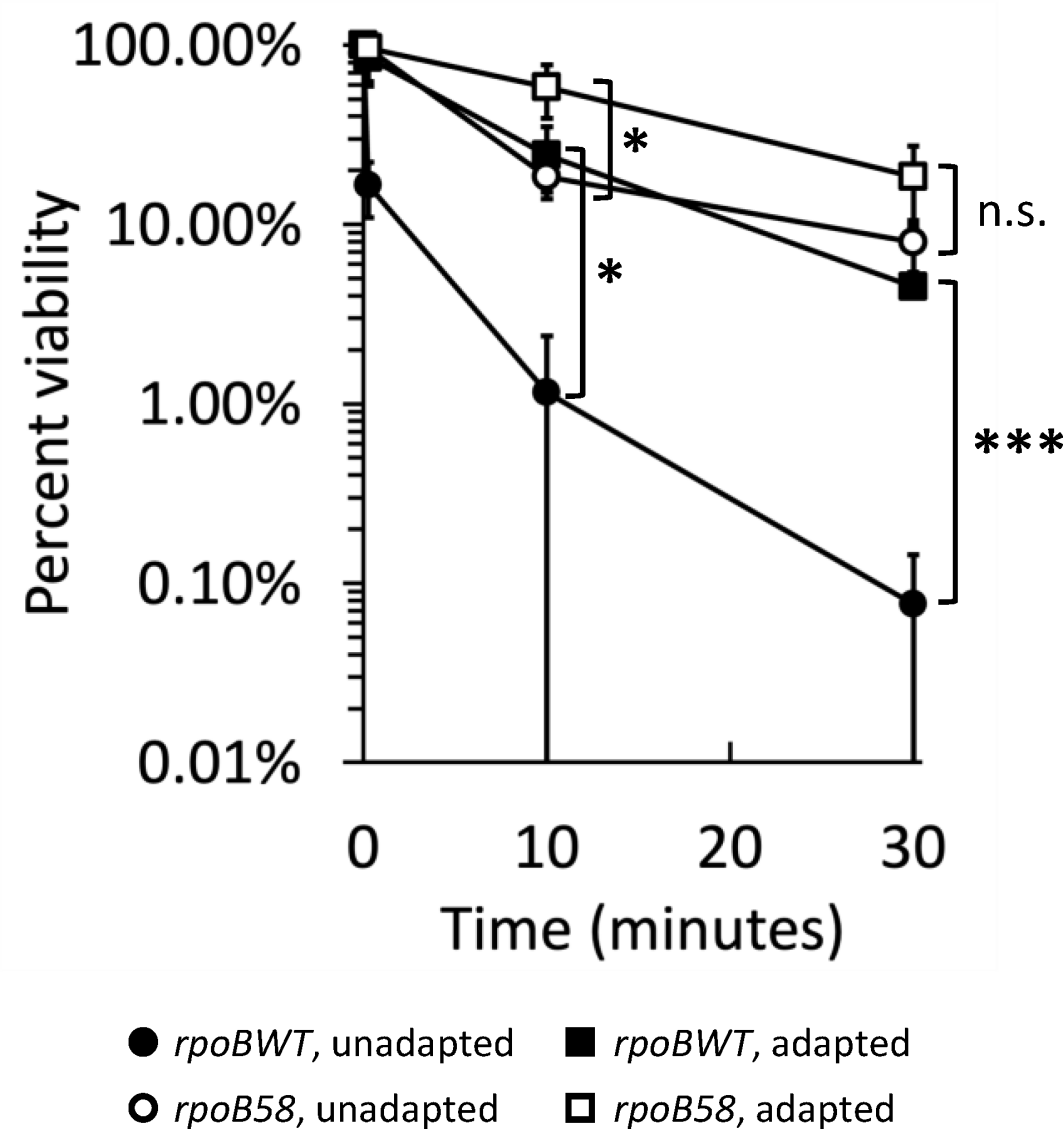
Pre-adaptation to mild shock confers tolerance to severe salt shock. RAM3027 (*rpoBWT*, solid symbols) and RAM3028 (*rpoB58*, open symbols) were grown to an OD_600_ of 0.2–0.3 in NB (no added NaCl) before pelleting and resuspending in either fresh NB (circles) or NB + 0.3 M NaCl (squares) to induce mild shock for 30 min. After 30 min, samples were shocked as in Fig. 1C to a final concentration of 1.3 M NaCl. The experiment was conducted in triplicate. Error bars indicate standard deviation. Statistically significant differences between unadapted and adapted pairs (*rpoBWT* pair, lower asterisks; *rpoB58* pair, higher asterisks) at each time point are marked with asterisks (n.s. not significant, *P < 0.05 and ***P < 0.001).

Osmotic shock tolerance can be conferred by pre-adapting cells to lower, non-lethal salt concentrations prior to shock (25). Both *rpoBWT* and *rpoB58* displayed increased osmotic shock tolerance upon pre-adaptation to 0.3 M NaCl, although the relative increase was greater in *rpoBWT* (Fig. 7). It is possible that pre-adaptation results in pre-emptive activation of SR or GSR, but the fact that *rpoB58* still benefits from pre-adaptation could indicate the involvement of additional shock tolerance mechanisms.

### Membrane damage is reduced in strains tolerant to hyperosmotic shock

**Fig 8:**
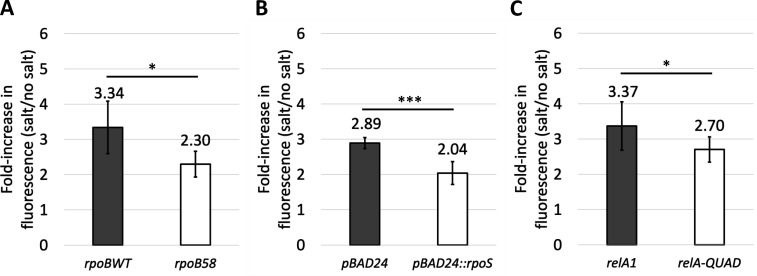
Uptake of propidium iodide (PI) is reduced in hyperosmotic shock-tolerant strains. (**A**) Strains RAM3027 (*rpoBWT*) and RAM3028 (*rpoB58*) were grown to mid-log (OD_600_ 0.3–0.4) in NB (no added NaCl) before inducing osmotic shock. (**B**) Strains RAM3743 (*rpoBWT* + pBAD24) and RAM3744 (*rpoBWT* + pBAD24*::rpoS*) were grown to mid-log (OD_600_ 0.3–0.4) in NB supplemented with ampicillin and 0.1% arabinose before inducing osmotic shock. (**C**) Strains RAM3027 (*rpoBWT relA1*) and RAM3486 (*rpoBWT relA^QUAD^*) were grown to mid-log (OD_600_ 0.3–0.4) in NB (no added NaCl) before inducing osmotic shock. Strains were grown as indicated before inducing osmotic shock by sudden upshift with NB + NaCl to 1.3 M, with or without PI present in the shock medium. After 30 min of shock, samples were washed and resuspended in M63 buffer before reading fluorescence. PI uptake was measured by excitation at 535 nm and emission at 615 nm. Fluorescence at 615 nm in PI-stained samples was normalized against unstained samples. Fold increase in fluorescence was calculated by dividing fluorescence of salt-shocked samples by fluorescence of unshocked samples. Error bars in each panel indicate standard deviation of values from five (**A and B**) and seven (**C**) independent cultures. Statistically significant differences are marked with asterisks (*P < 0.05 and ***P < 0.001).

Because osmotic stress can damage the membrane (15, 59), we examined loss of membrane integrity following osmotic shock. The DNA-binding dye propidium iodide (PI) is impermeable to intact membranes, and increased PI uptake has been used to demonstrate loss of membrane integrity (48, 60). Osmotic shocks were conducted with PI in the shock medium, and shocked cells were washed and examined for increased fluorescence, corresponding to PI uptake. We found that salt shock increased PI uptake, indicating negative effects on membrane integrity (Fig. 8). *rpoB58*, *relA^QUAD^*, and overexpression of *rpoS* all reduced PI uptake compared to controls following shock, indicating that these conditions confer osmotic shock tolerance at least in part by reducing membrane damage.

## DISCUSSION

Loss of viability during hyperosmotic shock likely results from catastrophic failure of multiple cellular structures and functions and detrimental effects on biophysical properties such as turgor pressure, all stemming from sudden water efflux immediately upon shock. Rapid cytoplasmic shrinkage from water loss increases macromolecular crowding (5), limiting macromolecular motion and diffusion-dependent reactions (7). Severe dehydration resulting from hyperosmotic shock damages structural properties of the inner membrane, resulting in phase separation and altered lipid packing, both of which could disrupt conformation and activity of membrane proteins (15, 61–64). The degree to which these general cellular properties are affected depends on the magnitude and abruptness of osmotic shock. *rpoBWT* cells experienced some immediate loss in viability upon moderate (i.e., 0.5 M NaCl) shock but almost fully recovered during the subsequent incubation period. However, during 0.75 or 1.3 M NaCl shock, the intrinsic osmotic shock tolerance mechanisms were overwhelmed, and *rpoBWT* cells were unable to recover viability in the shock period. In contrast, *rpoB58* cells not only experienced a much smaller immediate loss in viability upon 1.3 M shock but also maintained at least 10-fold better viability than *rpoBWT* throughout the incubation period. The shock-tolerant phenotype of unadapted *rpoB58* cells resembled that of *rpoBWT* cells pre-adapted to mild osmotic conditions (0.3 M NaCl). These observations suggest that *rpoB58* cells are in a “pre-adapted” physiological state of improved osmotic shock tolerance.

Several previous osmotic shock studies used modest NaCl concentrations ranging from 0.2 to 0.8 M NaCl (8, 21, 61, 62, 65–67). At these concentrations, plasmolysis was observed (8), but no loss of cell viability was reported (61, 62). The use of non-lethal salt concentrations during shock allowed the discovery of osmotolerance genes (29, 40, 66) and provided important details about compatible solutes and osmoadaptation (21). Unlike mild shock, challenges involving high salt concentrations of 1.3 M (this study) or up to 2.5 M (25) cause severe and sudden loss of cell viability, presumably because the basal levels of intrinsic defense mechanisms are inadequate and cells have no time to activate additional defense responses. However, the cells that survive the initial hyperosmotic shock do slowly recover, and we found that the recovery rate for *rpoBWT* and *rpoB58* cells is almost identical (Fig. 2C), indicating that *rpoB58*’s pre-emptive stress-tolerant state reduces initial loss of viability but does not improve subsequent recovery.

RNA-Seq data (34) indicated that *rpoB58* significantly alters the expression of genes in two stress-mitigating pathways, one of which is controlled by stationary phase general stress regulator RpoS and the other by SR regulator (p)ppGpp, the product of the RelA/SpoT proteins. We have shown that *rpoB58* bypasses the need for RpoS and RelA/SpoT and activates the stress-mitigating pathways even in their absence, which we hypothesize could play a role in the observed “pre-adapted” tolerant phenotype. *rpoB58* upregulated expression of many RpoS-controlled genes known to positively respond to mild osmotic shock, including *treA*, *osmE*, *osmY*, and *otsBA* (40), making them good candidates for conferring the hyperosmotic shock-tolerant phenotype of the mutant. However, individual deletions of these genes did not affect the *rpoB58* shock-tolerant phenotype. In fact, even deletion of *rpoS*, whose expression is induced by mild osmotic shock with 0.3 M NaCl (29) and constitutively elevated by *rpoB58* without shock (Fig. 4A and 5B; 34), had no effect on osmotic shock tolerance or sensitivity in either *rpoB* background. It is worth mentioning that disruption of *otsA* or *otsB* does confer osmotic sensitivity in glucose-minimal medium (23), but not in the NB medium used in this study. Similarly, *rpoS* has been shown to play a role in long-term survival of salt-stressed cells (68) but does not play a role in survival of the rapid, transient shock examined here.

Although most shock experiments here were performed at log phase, when *rpoS* expression is very low (Fig. 4A), we showed that plasmid overexpression of *rpoS* at log phase conferred osmotic shock tolerance, indicating that synthetic elevation of RpoS levels can confer shock tolerance to *rpoBWT* even in log phase. Consistent with the well-described stress-tolerant characteristics of stationary-phase cells (50, 51, 69, 70) and with reports connecting the stationary phase to osmotic shock tolerance (25, 71), we showed that stationary-phase cells are more tolerant to hyperosmotic shock than exponential-phase cells in both *rpoBWT* and *rpoB58* backgrounds, with both achieving similar levels of tolerance at the stationary phase. Surprisingly, stationary-phase shock tolerance was independent of RpoS in both *rpoB* backgrounds, indicating the involvement of RpoS-independent pathway(s) in osmotic shock tolerance.

SR is the second major stress pathway activated by *rpoB58* (34). The RpoS and SR regulons share many common genes (34, 41, 72), and RpoS synthesis is induced by SR determinants (p)ppGpp and DksA (36, 38, 73). We showed that *rpoB58* confers osmotic shock tolerance not only independent of RpoS, but also independent of RelA/SpoT, the two (p)ppGpp synthetases (56). Moreover, deletion of *relA* and *spoT* from the *rpoBWT* background did not further sensitize the strain to hyperosmotic shock. However, the presence of the constitutively active *relA^QUAD^* allele (57) and the overexpression of *relA* both elevated osmotic shock tolerance in *rpoBWT*. Furthermore, the *relA^QUAD^*-dependent increase in osmotic shock tolerance was independent of RpoS, indicating that activation of SR itself and not its subsequent activation of the RpoS regulon can cause osmotic shock tolerance.

The RpoS- and RelA/SpoT-independence of *rpoB58* osmotic shock tolerance can be explained by the fact that *rpoB58* constitutively activates the expression of genes belonging to the RpoS and SR regulons (34), thus pre-emptively readying the cell for various stresses, including hyperosmotic shock. *rpoBWT* can achieve a similar level of shock tolerance as *rpoB58* if pre-adapted to mild levels of NaCl (0.3 M). Similar osmosensitivity of *rpoBWT* cells with or without *rpoS* or *relA*/*spoT* indicates that merely having the capacity to trigger RpoS-mediated GSR or RelA/SpoT-mediated SR upon encountering stress is insufficient for conferring hyperosmotic shock tolerance. Instead, it appears that these regulons are only effective in conferring osmotic shock tolerance if pre-activated, either genetically or by pre-adaptation.

RelA/SpoT-mediated SR has been previously implicated in salt tolerance in *E. coli* (74, 75). A hallmark of both activated SR (72) and *rpoB58* (34) is the downregulation of genes involved in protein synthesis and upregulation of genes involved in the synthesis of certain amino acids. Since salt stress is known to destabilize protein folding (76), it is conceivable that lowering protein synthesis to rebalance protein homeostasis during salt stress could help cell survival. We recently showed that *rpoB58* confers extreme thermotolerance and molecular chaperone independence, driven in part by lowering protein synthesis (35). However, we found that inhibiting protein synthesis by sub-lethal chloramphenicol treatment decreased viability upon osmotic shock, rather than improving tolerance (data not shown). This suggests that the reduction of protein synthesis by activated SR and *rpoB58* is not likely responsible for the observed osmotic shock tolerance. Previous studies have shown that inhibition of protein synthesis by sub-lethal dosages of antibiotics or by removal of a ribosome maturation factor confers salt tolerance (74, 77). Critically, the authors measured salt tolerance by changes in optical density, rather than changes in colony-forming ability, noting that viability dropped significantly over the first hour of osmotic shock even in strains with reduced protein synthesis.

**Fig 9:**
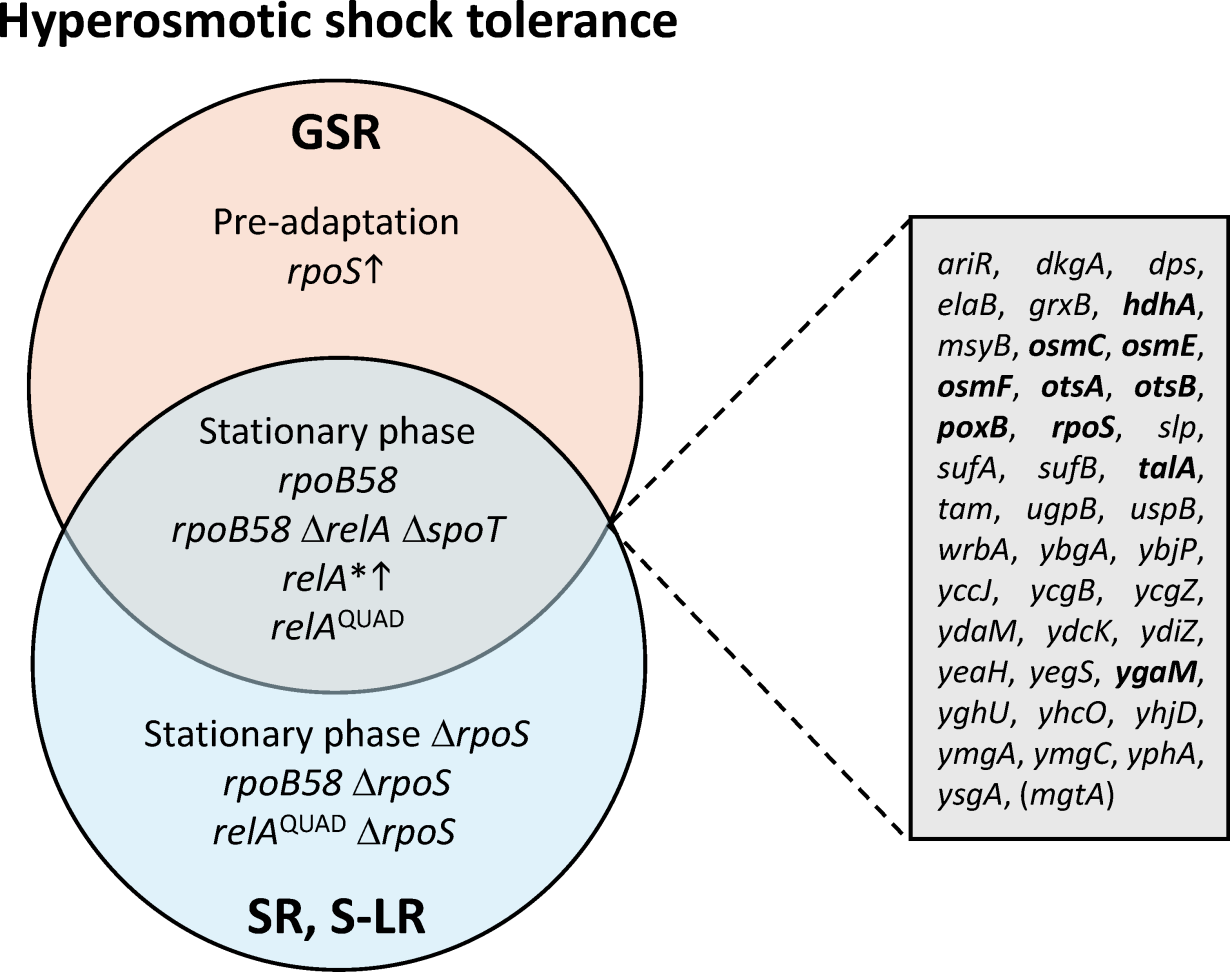
Shared changes in gene expression in overlapping conditions that confer hyperosmotic shock tolerance. Tolerance to extreme osmotic shock can be conferred by conditions that pre-activate GSR or SR/S-LR alone or by conditions associated with activation of both response pathways. Expression of 41 genes (boxed in the diagram) is commonly affected by GSR, SR, and *rpoB58*, of which 10 (bolded in the diagram) have previously been associated with osmotic stress tolerance. 40 genes are commonly upregulated, and one gene (*mgtA*, in parentheses) is commonly downregulated across all three responses.

The tolerance conditions examined here also reduced uptake of PI upon osmotic shock (Fig. 8), suggesting that the protective effects achieved independently by *rpoB58*’s stringent-like response (S-LR), SR, and GSR could commonly work by reducing membrane damage. While GSR is known to affect membrane structure and composition (78), the effects of SR and *rpoB58* on gene expression do not indicate that they cause significant changes to the membrane (34, 72). Furthermore, while *rpoB58* mimics aspects of both SR and GSR, it is still responsive to osmotic pre-adaptation, which could indicate either that *rpoB58* does not mimic the fullest extent of SR and GSR activation on osmotic shock tolerance or that additional shock tolerance pathways not pre-emptively activated by *rpoB58* are activated by pre-adaptation. A comparison of available RNA-Seq data shows that at least 40 genes are commonly upregulated across all three regulons, while 1 gene is commonly downregulated (Fig. 9). Among the upregulated genes, 10 genes (shown in bold) are implicated either directly (*otsA*, *otsB*, and *rpoS*) or indirectly (*hdhA*, *osmC*, *osmE*, *osmF*, *poxB*, *talA*, and *ygaM*) in osmotic stress response. However, disruption of *otsA*, *otsB,* or *rpoS* made no difference in *rpoB58* hyperosmotic shock tolerance, indicating that *rpoB58* may confer hyperosmotic shock tolerance by additional genes and pathways not common to all three stress responses or that have not previously been associated with osmotic stress tolerance.

## MATERIALS AND METHODS

### Strains, genetics, and growth methods

Most strains used in this work are a derivative of *E. coli* MC4100 (55). Strains and genotypes are listed in Table 1, and plasmids are listed in Table 2. Liquid cultures were grown in a roller drum at 37°C, and cultures on solid medium were incubated at 37°C for 18–24 h unless otherwise specified. Lysogenic broth (LB) Lennox (5 g/L NaCl) was prepared from Fisher LB Broth, Lennox powder. NB was prepared as salt-free complex medium from Difco NB powder. LB agar (LBA) plates were prepared with LB + 1.5% Difco agar from Becton Dickinson. When necessary, antibiotics were added (kanamycin [Km], 25 µg/mL; tetracycline [Tc], 10 µg/mL; chloramphenicol [Cm], 12.5 µg/mL; ampicillin [Ap], 50 or 100 µg/mL). Gene deletions were introduced via P1 phage transduction of selectable markers as described previously (79). When desired, the antibiotic resistance marker from the gene deletion site was removed using pCP20 (80).

**TABLE 1:** Bacterial strains used in this study

Strain name	Key genotype	Source
RAM1292	MC4100 (*relA1*) ∆*ara714*	(81)
RAM3027	RAM1292 *rpoBWT btuB*::Tn*10*	(34)
RAM3028	RAM1292 *rpoB58 btuB*::Tn*10*	(34)
RAM3133	MG1655 RLG4996 (*lacZ-lacY-rrnB* P1::*lacZ*)	(82)
RAM3185	AR3110 *synP8*::*lacZ*	(54)
RAM3188	RAM3185 *rpoBWT btuB*::Tn*10*	This work
RAM3189	RAM3185 *rpoB58 btuB*::Tn*10*	This work
RAM3337	RAM3027 ∆*queF*::Km^r^	(35)
RAM3338	RAM3027 *relA*^+^ ∆*queF*::Km^r^	(35)
RAM3341	RAM3028 ∆*queF*::Km^r^	(35)
RAM3406	RAM3027 ∆*queF*::scar	This work
RAM3407	RAM3028 ∆*queF*::scar	This work
RAM3486	RAM3027 *relA^QUAD^* ∆*queF*::Km^r^	This work
RAM3491	RAM3027 *relA^QUAD^* ∆*queF*::scar	This work
RAM3589	RAM1292 *relA*^+^ ∆*queF*::Km^r^	(35)
RAM3591	RAM3589 ∆*queF*::scar	(35)
RAM3595	RAM3591 *rpoBWT btuB*::Tn*10*	(35)
RAM3599	RAM3591 *rpoB58 btuB*::Tn*10*	(35)
RAM3657	RAM3595 ∆*relA*::Km^r^	(35)
RAM3661	RAM3599 ∆*relA*::Km^r^	(35)
RAM3665	RAM3657 *spoT*::Cm^r^	This work
RAM3669	RAM3661 *spoT*::Cm^r^	This work
RAM3741	RAM3027 ∆*rpoS*::Km^r^	This work
RAM3742	RAM3028 ∆*rpoS*::Km^r^	This work
RAM3743	RAM3027 + pBAD24	This work
RAM3744	RAM3027 + pBAD24::*rpoS*	This work
RAM3745	RAM3406 ∆*rpoS*::Km^r^	This work
RAM3746	RAM3407 ∆*rpoS*::Km^r^	This work
RAM3747	RAM3188 ∆*rpoS*::Km^r^	This work
RAM3748	RAM3189 ∆*rpoS*::Km^r^	This work
RAM3749	MG1655 RAM3133 + pALS13	This work
RAM3750	MG1655 RAM3133 + pALS14	This work
RAM3751	RAM3491 ∆*rpoS*::Km^r^	This work
RAM3752	RAM3027 ∆*otsA*::Km^r^	This work
RAM3753	RAM3038 ∆*otsA*::Km^r^	This work
RAM3754	RAM3027 ∆*otsB*::Km^r^	This work
RAM3755	RAM3028 ∆*otsB*::Km^r^	This work
RAM3756	RAM3027 ∆*osmY*::Km^r^	This work
RAM3757	RAM3028 ∆*osmY*::Km^r^	This work
RAM3758	RAM3027 ∆*osmE*::Km^r^	This work
RAM3759	RAM3028 ∆*osmE*::Km^r^	This work
RAM3760	RAM3027 ∆*treA*::Km^r^	This work
RAM3761	RAM3028 ∆*treA*::Km^r^	This work

**TABLE 2:** Plasmids used in this study

Plasmid	Description	Source
pBAD24	vector containing the P*_BAD_* promoter	(83)
pBAD24::*rpoS*	pBAD24 carrying the cloned *rpoS* gene	This work
pALS13	Contains a functional copy of the synthetase portion of the *relA* gene under the control of the P*_tac_* promoter	(58)
pALS14	Contains a truncated, non-functional fragment of the *relA* gene under the control of the P*_tac_* promoter	(58)

Due to a close P1 transductional linkage between *rpoS* and *relA*, ∆*rpoS* transductants were screened by colony PCR to confirm that appropriate *relA* alleles were maintained. *relA* alleles were transduced with the linked ∆*queF::Km* marker and confirmed by PCR screening. Primers used for *relA* screening were: forward 5′-AAGGAGAGGACGATGGTTGCG-3′, internal reverse 5′-TTCGGTTGGATGGAGGTAACGG-3′. A 2,100 bp product was expected for *relA1,* and 640 bp for *relA^WT^* or *relA^QUAD^*.

The *rpoS* overexpression plasmid was constructed by cloning *rpoS* onto a pBAD24 backbone, controlled by an arabinose-inducible promoter (83). The *rpoS* fragment was amplified from an MC4100 derivative bearing the wild-type *rpoS* allele using forward primer 5′-ACCTTCCATGGGTCAGAATACGCTG-3′ containing an NcoI site and reverse primer 5′-TTAAGCTTACTCGCGGAACAGCGCTTCG-3′ containing a HindIII site, and the product was cloned into the NcoI and HindIII sites of pBAD24.

### Osmotic shock assays

1-mL cultures were grown overnight in NB at 37°C, diluted 1:30 in fresh NB, and grown at 37°C to OD_600_ = 0.3–0.4 (approximately 2.5 h). Exponential-phase cultures were diluted 1:100 in fresh NB to 4 × 10^6^ CFU/mL. For each sample, 100 µL of diluted cells was mixed with 100 µL of pre-warmed treatment solution (NB or NB + 2.6 M NaCl unless otherwise specified) to obtain a final 200 µL solution of 2 × 10^6^ CFU/mL in NB or NB + 1.3 M NaCl. Samples were shocked at 37°C for 30 min, and 20-µL samples were withdrawn at 0 and 15 s and 10 and 30 min. Samples were serially diluted 10-fold in NB, and 10-µL aliquots were spotted onto LBA plates and incubated at 37°C overnight to allow colony formation. Viable cell counts from spots were used to calculate viability relative to time 0. Salt-shocked counts were normalized by dividing by NB-treated counts. Shock assays were conducted in triplicate unless otherwise specified.

For down-shock, strains were first subjected to the basic shock assay. After 10 min, three aliquots were withdrawn per sample and diluted in NB with no added salt or with 0.5 or 1.3 M NaCl. Dilutions were then plated onto LBA and incubated overnight to allow colony formation as described.

For *relA* overexpression, 1-mL cultures were grown overnight in NB + 100 µg/mL Ap at 37°C, diluted 1:30 in fresh NB + Ap, and grown at 37°C to OD_600_ = 0.2. IPTG was added to a final concentration of 10 µM, and cultures were grown for one additional hour before diluting 1:100 in NB for shock.

For *rpoS* overexpression, cultures were grown overnight in NB + 50 µg/mL Ap at 37°C, diluted 1:30 in fresh NB + Ap + 0.1% arabinose, and grown at 37°C to OD_600_ = 0.3–0.4. Cultures were then diluted 1:100 in NB for shock.

For pre-adaptation, 1-mL cultures were grown overnight at 37°C in NB, diluted 1:30 in fresh NB, and grown at 37°C to OD_600_ = 0.2–0.3. Two 1-mL samples of each culture were pelleted, resuspended in fresh NB or NB + 0.3M NaCl, and incubated standing at 37°C for 30 min. Cultures were then diluted 1:100 in the same medium (NB or NB + 0.3 M NaCl) before inducing shock, using NB with either 2.6 or 2.3 M NaCl to bring each sample to a final concentration of 1.3 M NaCl.

For growth phase shocks, cultures were grown in NB at 37°C either overnight (OD_600_ = 1.3–1.5) or to mid-log (OD_600_ = 0.3–0.4). Cultures were diluted to 4 × 10^6^ CFU/mL in NB before proceeding with the shock assay.

### Recovery assay

Strains were first subjected to the basic shock assay. After 10 min of shock, samples were diluted 10-fold into NB and incubated at 37°C for an additional 2 h. At indicated time points, samples were serially diluted 10-fold in NB and plated for incubation overnight as described.

### Beta-galactosidase assay

A strain was obtained containing the *synP8::lacZ* fusion, a construct in which a synthetic promoter exclusively dependent on RpoS activity was fused to *lacZ* (54). P1 lysates were prepared on strains containing *rpoB* alleles linked to *btuB::*Tn*10* and transduced into the fusion background. Strains were grown in LB to mid-log (OD_600_ 0.3–0.4) or stationary phase (overnight cultures, OD_600_ 4–5) before sampling for β-galactosidase assay by the method described previously (84). β-galactosidase activities were measured from at least three independent cultures in technical duplicate.

### Hydrogen peroxide sensitivity

Strains were grown in LB to stationary phase (overnight cultures, OD_600_ 4–5), pelleted by centrifugation, washed two times in 0.9% NaCl, and diluted in 0.9% NaCl to OD_600_ = 1.0. Suspensions were mixed with an equal volume of pre-warmed 0.9% NaCl or 0.9% NaCl + 30 mM H_2_O_2_ (final concentration 0 or 15 mM H_2_O_2_). To avoid decomposition of H_2_O_2_, treatment solutions were prepared fresh from 30% wt/wt stock solution just before assays. Samples were incubated at 37°C for 20 min before serially diluting 10-fold in 0.9% NaCl. 10-µL spots were plated on LBA and grown at 37°C overnight to allow colony formation.

### Propidium iodide uptake

PI stocks were prepared by dissolving powder in DMSO to 15 mM and diluting in water to 300 µM. Working stocks were diluted to 6 µM in shock media just prior to assays. Strains were grown as described, and 0.5 mL of culture was mixed with 0.5 mL of prewarmed media: NB or NB + 2.6 M NaCl, with or without PI. Final concentration of NaCl was 0 or 1.3 M, and final concentration of PI was 3 µM. 1-mL samples were shocked in a 37°C water bath for 30 min, then centrifuged at 13,000 × *g* to pellet the cells and remove the medium. Pellets were washed in M63 buffer, resuspended in 1 mL fresh M63, and transferred to quartz cuvettes. Fluorescence was read with a Varian Cary Eclipse spectrophotometer’s Scan application with settings: excitation range: 400–580 nm, emission: 615 nm, slits: 10 nm, sensitivity: high. 535 nm excitation and 615 nm emission wavelengths were used to report PI uptake. Fluorescence of PI-treated samples was normalized by dividing by the fluorescence of PI-free samples. Fold increase in uptake was determined by the dividing normalized fluorescence of salt-treated samples by no-salt samples.

### Statistical analysis

Two to seven biological replicates were used to calculate averages, standard deviations, and *P* values. Unpaired two-tailed Student *t*-tests were used to determine the statistical significance of differences between averages.
